# Diagnostic Challenges of Tumor Tissue and Circulating Microsatellite Status Assessment in Metastatic Colorectal Cancer and Their Impact on Access to Immunotherapy: A Real-World Retrospective Study

**DOI:** 10.3390/cancers18122006

**Published:** 2026-06-21

**Authors:** Benoist Chibaudel, Linda Dainese, Elisabeth Carola, Perrine Goyer, Hubert Richa, Arnaud Saget, Olivier Oberlin, Hélène Marijon, Nathalie Perez-Staub, Aimery de Gramont, Alain Toledano, Pascal Pujol

**Affiliations:** 1 Department of Medical Oncology, Hôpital Franco-Britannique—Fondation Cognacq-Jay, Cancérologie Paris Ouest, 92300 Levallois-Perret, France; helene.marijon@cognacq-jay.fr (H.M.); nathalie.perez-staub@cognacq-jay.fr (N.P.-S.); 2 Department of Pathology and Molecular Biology, IHP Group—Paris, 92240 Malakoff, France; ld@ihp-group.fr; 3 Department of Medical Oncology, Groupe Hospitalier du Sud de l’Oise, 60100 Creil, France; elisabeth.carola@ghpso.fr; 4 Department Digestive Surgery, Groupe Hospitalier Privé Ambroise Paré—Hartmann, 92100 Neuilly sur Seine, France; goyerperrine@gmail.com (P.G.); hubert.richa@cognacq-jay.fr (H.R.); dr.asaget@gmail.com (A.S.); docteur.oberlin@gmail.com (O.O.); 5 Department of Digestive Surgery, Hôpital Franco-Britannique—Fondation Cognacq-Jay, Cancérologie Paris Ouest, 92300 Levallois-Perret, France; 6 Cancérologie Paris Ouest, 92300 Levallois-Perret, France; aimerydegramont@gmail.com; 7 Department of Radiotherapy, Hartmann Oncology Radiotherapy Group, Cancérologie Paris Ouest, 92300 Levallois-Perret, France; alain.toledano@horg.fr; 8 Department of Genetic, Centre Hospitalier Universitaire de Montpellier, 34090 Montpellier, France; p-pujol@chu-montpellier.fr

**Keywords:** metastatic colorectal cancer, microsatellite instability, mismatch repair, diagnostic discordance, immunotherapy, real-world evidence

## Abstract

Background: Microsatellite instability (MSI) and mismatch repair (MMR) deficiency are key predictive biomarkers for immune checkpoint inhibitors (ICIs) in metastatic colorectal cancer (mCRC). In real-world practice, however, diagnostic pathways often involve heterogeneous testing modalities, which may lead to discordant or inconclusive results. Methods: We conducted a retrospective study of patients with mCRC who underwent at least one MSI/MMR assessment between 2015 and 2025. Diagnostic modalities included IHC, tissue-based and liquid-based MSI testing. A predefined decision algorithm classified results as conclusive or inconclusive; discordant cases underwent adjudication that integrated a pathology review, molecular features, and technical considerations. Patients were ultimately assigned to definitive MSS or definitive MSI groups. Clinical characteristics, treatment patterns, and outcomes—particularly in relation to immunotherapy—were evaluated. Results: Among 727 evaluable patients, the MSI/MMR status was conclusive in 695 (95.6%) and inconclusive in 32 (4.4%). Inconclusive cases resulted from isolated MMR protein loss, heterogeneous or equivocal staining, inter-tumoral discordance, or discrepancies between tissue- and liquid-based assays. After adjudication, 54 patients (7.4%) were classified as definitive MSI and 673 (92.6%) as definitive MSS. Definitive MSI tumors were associated with female sex, right-sided primaries, high-grade histology, nodal involvement, and BRAF V600E mutations. Among the definitive MSI patients, 31 (57.4%) received immunotherapy, achieving a complete response rate of 48.4% and an overall response rate of 71.0%. Median PFS and OS were not reached in the definitive MSI group, whereas definitive MSS patients treated with ICIs experienced significantly poorer outcomes. Conclusive and adjudicated MSI groups demonstrated comparable responses to immunotherapy. Conclusions: In real-world practice, a meaningful proportion (4%) of mCRC patients experience inconclusive MSI/MMR assessment, with important clinical implications. Both technical and biological factors contribute to diagnostic uncertainty. Integrating orthogonal testing modalities and applying structured adjudication improves classification accuracy and ensures appropriate access to immunotherapy.

## Simple Summary

Accurate identification of tumors with microsatellite instability is essential for selecting patients with metastatic colorectal cancer who may benefit from immunotherapy. In clinical practice, however, patients often undergo several different diagnostic tests performed on either tissue or blood, and these methods do not always yield consistent results. Such discrepancies may arise from technical limitations, variable tumor biology, or differences in how each assay detects genomic instability. This study evaluated how frequently these inconsistencies occur, explored their underlying causes, and examined their impact on treatment decisions. By systematically reviewing discordant cases and integrating information from multiple diagnostic platforms, we were able to clarify most uncertain findings. Overall, our results highlight the importance of a multimodal diagnostic approach to improve the accuracy of mismatch repair and microsatellite instability assessment. This strategy can help ensure that patients most likely to benefit from immunotherapy are correctly identified and appropriately treated.

## 1. Introduction

Microsatellite instability (MSI) and mismatch repair (MMR) deficiency (dMMR) represent essential biomarkers in metastatic colorectal cancer (mCRC), predicting profound and durable responses to immune checkpoint inhibitors (ICIs) [1,2]. Since the approval of pembrolizumab and nivolumab for MSI-high/dMMR tumors, accurate determination of MSI/MMR status has become a critical step in therapeutic decision-making [3,4,5,6].

Multiple diagnostic modalities exist, including immunohistochemistry (IHC); PCR-based MSI testing; next-generation sequencing (NGS); genomic signatures; and more recently, liquid-based assays such as circulating tumor DNA (ctDNA). While concordance between methods is generally high, real-world testing is often challenged by sample quality, tumor heterogeneity, and technical limitations [7,8,9,10]. Consequently, some patients receive discordant or inconclusive results. Such discrepancies raise significant challenges, as they may lead to misclassification of patients and inappropriate exclusion from potentially effective immunotherapy.

Discordance in MMR assessment may arise from technical factors, such as pre-analytical variability, antibody performance, tumor cellularity, or analytical thresholds [11,12,13]. These issues are, in principle, avoidable through methodological optimization and standardization. However, discordance may also reflect underlying biological mechanisms, including intratumoral heterogeneity, clonal evolution under treatment pressure, or differential shedding of tumor DNA into the bloodstream [12,13,14]. Understanding whether a discordant result is technical or biologically driven is therefore crucial for accurate interpretation.

Given the binary nature of current MSI/MMR classification (pMMR/MSS versus dMMR/MSI-high), clinicians are often faced with complex decision making when confronted with inconsistent results across platforms. Clarifying the mechanisms behind discordance may optimize diagnostic pathways, enhance MSI/MMR reliability, and refine immunotherapy selection. The frequency, causes, and clinical consequences of inconclusive MSI/MMR assessments were analyzed, with a particular focus on the subgroup experiencing diagnostic uncertainty.

## 2. Materials and Methods

### 2.1. Study Objectives

In this study, we described different diagnostic testing modalities for MSI/MMR status assessment, including IHC, molecular testing, circulating MSI signature and comprehensive genomic profiling (CGP), in a cohort of patients with mCRC. We analyzed the frequency, nature, and potential causes of discordance across methods, and evaluated their clinical implications, particularly regarding eligibility for immunotherapy. Our findings aim to provide clinicians with practical insights to better interpret discordant MSI/MMR results and optimize therapeutic decisions in mCRC.

### 2.2. Study Design and Population

This retrospective study included all consecutively seen patients at our institution with histologically proven metastatic colorectal cancer and undergoing MSI/MMR testing between January 2015 and December 2025.

Eligible patients were required to have undergone at least one assessment of MSI/MMR on tumor tissue and/or liquid biopsy during the course of their disease. Individual-level clinical, pathological, and molecular data were extracted from the institutional electronic medical record system (DxCare software, version 8.2021.2.8, Dedalus, Antony, France).

### 2.3. Diagnostic Modalities

Testing modalities included tissue-based and/or liquid-based testing. Tissue-based MMR and MSI statuses were defined using IHC and molecular testing, respectively. IHC for the four MMR proteins (MLH1, PMS2, MSH2, MSH6) was performed according to local pathology laboratory procedures. Molecular testing was performed either by PCR-based MSI analysis or next generation sequencing (NGS), depending on the methodology routinely used by the testing platform. The thresholds used to classify MSS and MSI-high were aligned with the standard criteria established for each method.

Circulating tumor DNA (ctDNA) analysis was performed using commercially available assays, including FoundationOne^®^ Liquid CDx (Foundation Medicine, Inc., Boston, MA, USA) or Guardant360 CDx (Guardant Health, Inc., Palo Alto, CA, USA), according to manufacturer specifications. Liquid-based testing results were reported as ctMSI-high or ctMSS.

### 2.4. Diagnostic Rules and Classification

Assessment of MSI/MMR status followed a predefined decision algorithm integrating all available testing modalities, including IHC, tissue-based testing, and liquid-based testing when applicable. For each patient, results were first evaluated for internal consistency and diagnostic clarity. Tests showing unequivocal results—either intact expression of all MMR proteins, complete loss of common paired MMR proteins (MLH1/PMS2 or MSH2/MSH6) or MSI-high molecular status—were classified as conclusive and further categorized as conclusive MSS or conclusive MSI. In contrast, any case presenting diagnostic uncertainty, including atypical IHC patterns (e.g., isolated loss of MMR protein expression, uncommon pairing), suboptimal assay quality, discordant finding between tumor sites, or discordant findings between IHC and molecular testing, was classified as inconclusive. Discordant cases underwent retrospective adjudication based on pathology review, technical considerations, and molecular features such as MMR gene mutations or germline testing. The adjudication process was conducted retrospectively and was blinded to clinical data, treatment information and patient outcomes.

Based on the final integrated interpretation, patients were assigned to one of two definitive groups: a definitive MSS group, comprising both conclusive MSS and adjudicated MSS cases, and a definitive MSI group, comprising conclusive MSI and adjudicated MSI cases.

### 2.5. Data Collection

For each patient, demographic, clinical, pathological, and technical variables were collected from the electronic medical record, including age, sex, tumor characteristics (primary tumor sidedness, histological grade, initial stage, metastatic sites), and molecular features (*KRAS*, *NRAS*, *BRAF* gene mutational status). Treatment data—including exposure to ICI and tumor response, as assessed by the treating physician—were also analyzed.

### 2.6. Statistical Analysis

Continuous variables were summarized using means and standard deviations (SDs), while categorical variables were reported as frequencies and percentages. Comparisons of baseline characteristics between groups were performed using the chi square test or Fisher’s exact test for categorical variables, and the Mann–Whitney U test for continuous variables, as appropriate.

For patients who received ICIs, survival outcomes included progression-free survival (PFS) and OS from ICI initiation. PFS from ICI was defined as the time from initiation of ICI to documented disease progression or death from any cause. OS from ICI was defined as the time from initiation of ICI to death from any cause. Patients without an event at the time of data cutoff were censored at the date of last follow up. Survival curves were estimated using the Kaplan–Meier method, and differences between groups were assessed using the log rank test.

Comparisons between patients with definitive MSI and definitive MSS receiving immunotherapy were conducted using descriptive statistics only, as these exploratory analyses were intended to illustrate observed clinical patterns without performing multivariable adjustment or propensity-based methods.

All statistical analyses were performed using GraphPad Prism software (MedCalc Software Ltd., version 22.021, Ostend, Belgium; https://www.medcalc.org, accessed on 17 June 2026). A two-sided *p*-value < 0.05 was considered statistically significant.

## 3. Results

### 3.1. Study Population

**Figure 1:**
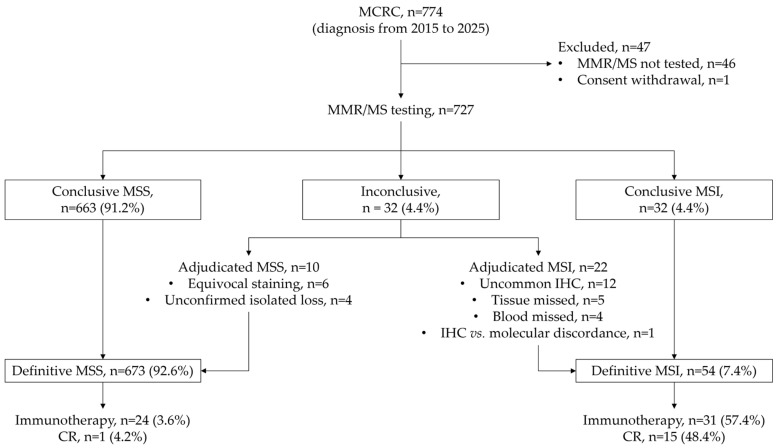
Flow diagram. Abbreviations: MCRC, metastatic colorectal cancer; MMR, mismatch repair; MS, microsatellite; MSS, microsatellite stable; MSI, microsatellite instability; IHC, immunohistochemistry; CR, complete response.

Among 774 patients with histologically proven mCRC, 727 were included in the final analysis after exclusion of 46 patients with missing or unavailable MSI/MMR testing results and one patient who declined consent for data use (Figure 1).

### 3.2. Patient Characteristics

The study population had a mean age of 66.7 years (range 23.9 to 97.7), and 51.2% were men. A right-sided primary tumor was identified in 33.4% of patients, and 11.6% of tumors were classified as poorly differentiated. Most patients presented with synchronous metastatic disease (65.3%), and metastatic involvement was limited to a single organ at diagnosis in 65.5% of cases. The liver was the most frequent metastatic site, involved in 64.2% of patients. Regarding molecular features, RAS-mutated tumors accounted for 55.4% of cases, whereas BRAF-mutated tumors represented 8.9% (Table 1).

### 3.3. MSI/MMR Testing

#### 3.3.1. Testing Modalities

A total of 403 patients underwent tissue-only testing, 12 underwent liquid-only testing, and 312 had paired tissue and blood analyses.

Tissue-based assessment was performed in 715 patients (98.3%), using IHC alone in 633 patients (88.5%), a molecular assay alone in 24 patients (3.4%), and both modalities in 58 patients (8.1%). The three most commonly used molecular testing methods were Idylla MSI (*n* = 54), Pentaplex (*n* = 9), and FoundationOne Tissue (*n* = 4). Various other techniques were applied in seven patients, and the testing method was not documented for nine patients. Notably, one patient underwent both Idylla and Pentaplex MSI testing.

A liquid biopsy was obtained in 310 patients (42.6%), using FoundationOne^®^Liquid in 257 (82.9%) patients, Guardant360 CDx in 42 cases (13.5%), or both assays in 11 cases (3.5%). Liquid biopsies were non-informative in 12 patients (3.9%). Germline testing of MMR genes was performed in 44 patients (6.1%).

#### 3.3.2. Testing Results

MSI/MMR results were conclusive in 695 patients (95.6%) and inconclusive in 32 patients (4.4%). Among the 663 patients classified as conclusive MSS, 628 exhibited a pMMR phenotype, 24 were MSS by molecular testing, and 11 were ctMSS. Among the 32 patients with conclusive MSI status, 31 displayed typical MMR-deficient patterns, characterized by loss of MLH1/PMS2 (*n* = 27) or MSH2/MSH6 (*n* = 4). In these patients, additional molecular MS testing was performed in 18 cases, all of which confirmed an MSI-high status. MSI was identified exclusively through liquid biopsy in one patient who had no prior tissue-based MSI/MMR assessment. The 32 inconclusive cases encompassed a range of diagnostic challenges (Table 2, Appendix A).

Inconclusive IHC findings were identified in 23 patients (3.2%; Appendix A, ID 1 to 23), predominantly due to uncommon IHC patterns in 22 cases—comprising 15 instances of isolated MMR protein loss (including three cases with discrepant results between tumor sites), six cases of equivocal or heterogeneous staining, and one case with an atypical protein-loss pairing—and one additional patient showing IHC–molecular discordance (pMMR/MSI-high). Among the 15 patients with isolated MMR protein loss, PMS2 was involved in seven patients, MSH6 in six patients and MLH1 in two patients. Further molecular testing was performed in 13 patients, yielding 10 adjudicated MSI and three adjudicated MSS classifications. Two patients with isolated MLH1 loss were considered adjudicated MSI without molecular confirmation. All six patients with equivocal staining were adjudicated MSS after IHC review, retesting or further MSS molecular diagnosis. Among the three patients with discrepant MMR profiles between primary and metastatic sites, two were adjudicated MSI. The single patient with an uncommon MLH1/MSH6 loss pattern was adjudicated MSI following molecular testing.

Discrepancies between tissue-based and liquid-based testing were observed in nine patients (Appendix A, IDs 24 to 32), including six with tMSS/ctMSI and three with tMSI/ctMSS; all were ultimately adjudicated MSI.

Of the 32 patients with inconclusive results, 22 were classified as adjudicated MSI and 10 as adjudicated MSS. Based on the final integrated classification, 673 patients (92.6%) were assigned to the definitive MSS group and 54 patients (7.4%) to the definitive MSI group. The definitive MSI cohort showed associations with female patients, right-sided primaries, high-grade tumors, nodal involvement and *BRAF* V600E mutations, whereas definitive MSS tumors correlated with hepatic involvement and *RAS*-mutant status (Table 1).

### 3.4. Access to Immunotherapy and Clinical Outcomes

Of the 54 patients with definitive MSI tumors, 31 (57.4%) received ICIs, including five (16.1%) treated between 2015 and 2020 and 26 (83.9%) treated between 2021 and 2025. ICIs were given in 26 (83.9%) patients as first-line setting, including 21 patients who received a PD-1 or PDL-1 inhibitor single agent, four patients received the combination of PD-1 and CTLA4 inhibitors, and one patient received a 3-month induction triplet chemotherapy followed by maintenance therapy with PD-1 inhibitor. Among the five patients who received ICIs as a second- or later-line setting, four patients received PD1 or PDL1 inhibitor as a single agent, and one patient received PD-1 inhibitor followed by a combination of PD-1 and CTLA4 inhibitor.

#### 3.4.1. Definitive MSI vs. Definitive MSS Groups

ICIs were administered to 31 patients (57.4%) in the definitive MSI group and to 24 patients (3.6%) in the definitive MSS group. Notably, immune checkpoint inhibitors were combined with chemotherapy in the latter group (Table 3).

A complete response was achieved in 15 patients (48.4%) in the definitive MSI group and none in the definitive MSS group (*p* = 0.0001). The overall response rates were 71.0% and 29.2% in the definitive MSI and definitive MSS groups, respectively (*p* = 0.002).

**Figure 2:**
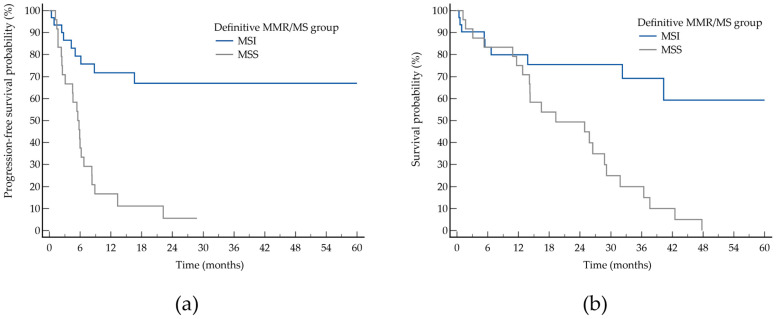
Progression-free survival from ICI (**a**) and survival from ICI (**b**) were estimated from starting immunotherapy according to definitive MSI/MMR groups (definitive MSI: blue solid line, *n* = 31 vs. definitive MSS: grey solid line, *n* = 24).

The median follow-up from the initiation of immunotherapy was 39.5 months (95% CI, 25.3–87.0). Median PFS was not reached in the definitive MSI group, whereas it was 5.6 months (95% CI, 2.5–6.7) in the definitive MSS group (HR 0.19, 95% CI, 0.09–0.40; *p* < 0.0001) (Figure 2a). Similarly, median OS was not reached in the definitive MSI group and was 19.3 months (95% CI, 12.8–47.8) in the definitive MSS group (HR 0.26, 95% CI, 0.13–0.55; *p* = 0.0004) (Figure 2b).

In patients with definitive MSI tumors, median OS was not reached in those who received immunotherapy (*n* = 31), compared with 13.7 months in those who did not (*n* = 23) (HR 0.30, 95% CI 0.14–0.67; *p* = 0.003).

#### 3.4.2. Conclusive MSI vs. Adjudicated MSI

**Figure 3:**
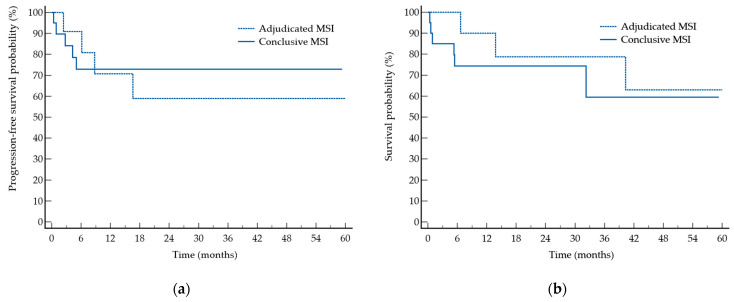
Progression-free survival from ICI (**a**) and survival from ICI (**b**) were estimated from starting immunotherapy in “conclusive MSI” (blue solid line, *n* = 20) and “adjudicated MSI” groups (blue dashed line, *n* = 11).

Immunotherapy was administered to 20 patients (62.5%) in the conclusive MSI group and to 11 patients (50.0%) in the adjudicated MSI group. A complete response was achieved in eight patients (40.0%) and seven patients (63.6%) in the conclusive MSI and adjudicated MSI groups, respectively (*p* = 0.215). The ORRs were 70.0% and 72.7% in the conclusive MSI and adjudicated MSI groups, respectively (*p* = 0.875). Median PFS from the start of immunotherapy was not reached in either group (HR 1.25, 95% CI 0.33–4.77; *p* = 0.744) (Figure 3a). Similarly, median OS was not reached in either group (HR 1.25, 95% CI 0.61–2.35; *p* = 0.472) (Figure 3b).

#### 3.4.3. Adjudicated MSS Tumors

Among the 10 patients adjudicated as having MSS tumors, two exhibited an atypical dMMR profile with isolated loss of PMS2 expression on IHC; however, subsequent molecular testing failed to confirm MSI-high status, instead classifying one tumor as MSS and the other as MSI-low. Both patients received immunotherapy—one in the first-line setting with pembrolizumab and one in the third-line setting with nivolumab–ipilimumab—and each experienced disease progression as best response.

## 4. Discussion

This study highlights that diagnostic challenges in determining MSI/MMR status in mCRC are not uncommon, with an overall frequency of 4%. Interestingly, this proportion mirrors the prevalence of tumors initially classified as MSI in our cohort, underscoring the clinical relevance of identifying and resolving MSI/MMR diagnostic uncertainty. Among the 32 patients with inconclusive results, 69% (22 patients) were ultimately adjudicated as MSI, demonstrating that discordant or ambiguous findings frequently conceal biologically meaningful MMR deficiency or microsatellite instability.

The underlying causes of inconclusive testing were heterogeneous and strongly influenced the likelihood of reclassification. Equivocal IHC staining invariably led to a final MSS interpretation (4/4), suggesting that such patterns predominantly reflect technical or interpretative limitations rather than true MMR deficiency. In contrast, isolated loss of a single MMR protein—a well-recognized but uncommon scenario—was associated with a high probability of MSI upon further evaluation, with 11 out of 14 cases ultimately adjudicated as MSI. These findings reinforce the need for systematic reassessment of isolated MMR protein loss using complementary molecular assays, particularly in metastatic disease, where therapeutic implications are substantial.

In this series, isolated loss of MMR protein expression was the most frequent cause of inconclusive results and was observed in 15 of the 691 tumors assessed by IHC, corresponding to an overall frequency of 2.2%. Isolated loss of a single MMR protein on IHC can arise from several biological and technical mechanisms. First, germline or somatic mutations affecting the partner protein in the heterodimer (e.g., MLH1–PMS2 or MSH2–MSH6) may destabilize only one component, leading to selective loss of expression despite intact function of the remaining partner [15,16,17]. Second, epigenetic alterations—most commonly MLH1 promoter hypermethylation—can produce partial or discordant protein loss, particularly in tumors with heterogeneous methylation patterns [18,19,20,21]. Third, technical artifacts related to tissue fixation, antigen retrieval, or antibody performance may generate false-positive isolated loss, especially for PMS2 and MSH6, which are more sensitive to pre-analytical variability [22,23,24,25]. Fourth, subclonal or heterogeneous dMMR within the tumor may result in patchy staining patterns that mimic isolated loss but do not reflect a fully MSI-high molecular phenotype [26,27]. Finally, non-pathogenic variants or low-penetrance alterations can impair protein stability without producing sufficient microsatellite instability to meet MSI-high thresholds on molecular assays [28]. Together, these mechanisms underscore the importance of integrating IHC with orthogonal molecular testing to avoid misclassification of tumors with atypical dMMR profiles.

Molecular MSI assays based on PCR or NGS also have inherent constraints, including panel-dependent sensitivity, since instability may occur in loci not represented in standard microsatellite panels or at levels below assay detection thresholds [29,30,31]. This limitation was illustrated in our series by a patient with an IHC dMMR phenotype showing isolated PMS2 loss, for whom molecular testing yielded discordant results: MSS using the Biocartis Idylla assay but MSI-high with the Pentaplex method. Finally, MSI detection in circulating tumor DNA is challenged by several mechanisms that underlie discordant results, such as heterogeneous tumor biology, unequal shedding from primary versus metastatic lesions, asynchronous collection of tissue and plasma, and inherently low ctDNA release in lung- or peritoneum-limited metastatic disease [32]. Several technical and biological factors can also influence the performance of ctDNA-based MSI testing, including the tumor fraction, assay sensitivity, timing of blood collection, and overall tumor burden. These factors highlight the importance of integrating multiple complementary approaches and interpreting atypical results within the broader clinical and pathological context.

Access to immunotherapy was achieved in 57% of patients with definitive MSI tumors, with no difference between those classified as MSI at initial testing and those reclassified after adjudication. This relatively modest uptake must be interpreted in the context of the study period (2015–2025). In Europe, regulatory approval for PD-1 inhibitors in metastatic colorectal cancer was granted only in January 2021 by the European Medicines Agency, and reimbursement in France was implemented in June 2021. These timelines inevitably limited early access to immunotherapy, particularly for patients diagnosed before 2021. Despite these constraints, the clinical benefit of immunotherapy observed in our MSI population aligns closely with outcomes reported in pivotal trials [33,34,35,36,37]. Importantly, the magnitude of benefit was comparable between patients with an initial MSI classification and those with an MSI status established only after adjudication. This finding emphasizes that patients with adjudicated MSI tumors should be considered equally eligible for immunotherapy, provided that diagnostic uncertainty is adequately resolved through a structured, multimodal testing algorithm. A potential source of selection bias in the comparison of OS between MSI patients who did and did not receive immunotherapy relates primarily to differences in treatment availability over time. Consequently, patients who received ICIs were more likely to have been diagnosed or treated in more recent years, whereas immunotherapy-naive MSI patients disproportionately reflect earlier periods when ICIs were not yet accessible. This temporal imbalance may influence observed survival differences independently of treatment effect.

The markedly higher response rates and survival outcomes observed in the definitive MSI group reinforce the strong sensitivity of MSI-H/dMMR tumors to immune checkpoint blockade. Nearly half of the MSI patients achieved a complete response, and both PFS and OS were not reached, underscoring the depth and durability of benefit typically associated with immunotherapy in this molecular subgroup. In contrast, patients with definitive MSS tumors—who received ICIs only in combination with chemotherapy—experienced substantially lower response rates and significantly shorter PFS and OS, consistent with the limited activity of ICIs in MSS colorectal cancer. These exploratory comparisons between definitive MSI and definitive MSS patients should be interpreted with caution, as the analyses were descriptive in nature and may be subject to confounding due to the small MSI sample size and heterogeneity of the cohort. Overall, the results align with existing evidence supporting ICIs as a highly effective treatment option for MSI-H/dMMR metastatic colorectal cancer, while highlighting the persistent unmet need for effective immunotherapy strategies in MSS disease.

Our results illustrate the increasing complexity of MSI/MMR assessment in the era of expanding diagnostic modalities. The availability of multiple tumor-based and circulating assays introduces variability but simultaneously enhances diagnostic sensitivity. In our cohort, this broader testing landscape enabled the identification of additional MSI tumors that would otherwise have remained undetected, thereby increasing the number of patients eligible for immunotherapy—a therapeutic strategy with a profound impact on overall survival.

## 5. Conclusions

In routine clinical practice, a subset of mCRC patients experience inconclusive MSI/MMR testing. Improving diagnostic pathways is essential to ensure equitable treatment opportunities. These findings support the need for standardized diagnostic algorithms, including reflex testing, mandatory second-line assays for ambiguous cases, and integration of ctDNA-based MSI testing when tissue is limited.

## Figures and Tables

**Table 1:** Patient characteristics, *n* = 727.

Covariate	Class	All	Definitive MSS	Definitive MSI	*p*-Value
Age	<70	444 (61.1)	417 (62.0)	27 (50.0)	0.083
	≥70	283 (38.9)	256 (38.0)	27 (50.0)	
Sex	Female	355 (48.8)	320 (47.5)	35 (64.8)	0.015
	Male	372 (51.2)	353 (52.5)	19 (35.2)	
Primary tumor sidedness	Left-sided	477 (65.6)	458 (68.1)	19 (35.2)	<0.001
	Right-sided	243 (33.4)	208 (30.9)	35 (64.8)	
	Both	7 (1.0)	7 (1.0)	0	
Grading	Low	540 (74.3)	510 (75.8)	30 (55.6)	<0.001
	High	84 (11.6)	65 (9.7)	19 (35.2)	
	Missing	103 (14.2)	98 (14.6)	5 (9.3)	
Initial stage	Non-metastatic	252 (34.7)	228 (33.9)	24 (44.4)	0.117
	Metastatic	475 (65.3)	445 (66.1)	30 (55.6)	
No of metastatic sites	1	476 (65.5)	436 (64.8)	40 (74.1)	0.167
	>1	251 (34.5)	237 (35.2)	14 (25.9)	
Organ involvement	Liver	467 (64.2)	447 (66.4)	20 (37.0)	<0.001
	Lung	195 (26.8)	185 (27.5)	10 (18.5)	0.153
	Node	126 (17.3)	103 (15.3)	23 (42.6)	<0.001
	Peritoneum	200 (27.5)	185 (27.5)	15 (27.8)	0.964
RAS/BRAF ^1^ status	Wild-type	208 (28.6)	191 (28.4)	17 (31.5)	<0.001
	RAS mutant	403 (55.4)	388 (57.7)	15 (27.8)	
	BRAF mutant	65 (8.9)	48 (7.1)	17 (31.5)	
	Missing	51 (7.0)	46 (6.8)	5 (9.3)	

^1^ *KRAS* exons 2–4, *NRAS* exons 2–4 and *BRAF* V600E mutational status.

**Table 2:** Type of discordant cases, definitive adjudicated profile and adjudication process.

Type of Discordance	No of Cases	Adjudicated MSI	Adjudication Process
All types	32	22	
Tissue discordance			
Isolated loss of MMR protein	15	11	
MLH1	2	2	Adjudicated MSI: considered as dMMR-IHC with no further test
MSH2	0	N/A	
MSH6	6	4	Adjudicated MSS: unconfirmed loss of MSH6 expression after IHC second review or retesting Adjudicated MSI: molecular MSI-high (including 2 cases of Lynch syndrome)
PMS2	7	5	Adjudicated MSS: MSS or MSI-low Adjudicated MSI: molecular MSI-high
Equivocal or heterogeneous staining (IHC)	6	0	Adjudicated MSS: unconfirmed staining after IHC second review or retesting (*n* = 3), molecular MSS or ctMSS (*n* = 3)
Uncommon IHC pairing	1	1	Adjudicated MSI: considered as dMMR-IHC with no further test
pMMR/MSI-high	1	1	Adjudicated MSI: molecular MSI-high (Pentaplex 5/5)
Discordance between tissue and blood			
Blood missed (tissue dMMR and MSI-high/ctMSS)	3	3	Adjudicated MSI: concordant tissue testing (IHC and molecular), interval between tissue testing and liquid biopsy (temporal heterogeneity)
Tissue missed (tissue pMMR/ctMSI-high)	6	6	Adjudicated MSI: circulating MSI signature positive (*n* = 1), MMR mutated gene using circulating CGP (*n* = 3), germline MMR gene mutation (*n* = 2)

**Table 3:** Immunotherapy access and response rate according to MSI/MMR group, *n* (%). Abbreviations: MSI, microsatellite instability; MSS, microsatellite stable; N, number of patients; CR, complete response; PR, partial response; SD, stable disease; PD, progressive disease; NE, not evaluable; ORR, overall response rate; DCR, disease control rate. Background color was used to illustrate that those columns are the sum of the white ones.

Clinical Outcome	Definitive MSI	Conclusive MSI	Adjudicated MSI	Definitive MSS	Conclusive MSS	Adjudicated MSS
N	54	32	22	673	663	10
Immunotherapy	31 (57.4)	20 (62.5)	11 (50.0)	24 (3.6)	22 (3.3)	2 (20.0)
CR	15	8	7	0	0	0
PR	7	6	1	7	7	0
SD	4	2	2	9	9	0
PD	4	3	1	8	6	2
NE	1	1	0	0	0	0
ORR (CR+PR)	22 (71.0)	14 (70.0)	8 (72.7)	7 (29.2)	7 (31.8)	0 (0.0)
DCR (CR+PR+SD)	26 (83.9)	16 (80.0)	10 (90.9)	16 (66.7)	16 (80.0)	0 (0.0)

## Data Availability

The data supporting the findings of this study are available on request from the corresponding author. The data are not publicly available due to their containing information that could compromise the privacy of research participants.

## Abbreviations

The following abbreviations are used in this manuscript:

MSI	Microsatellite instability
MMR	Mismatch repair
dMMR	MMR deficient
pMMR	MMR proficient
CRC	Colorectal cancer
mCRC	Metastatic colorectal cancer
ICI	Immune checkpoint inhibitor
MS	Microsatellite
MSS	Microsatellite stable
IHC	Immunohistochemistry
PCR	Polymerase chain reaction
NGS	Next generation sequencing
ct	Circulating tumor
ctDNA	Circulating tumor DNA
MLH1	MutL Homologue 1
MSH2	MutS Homologue 2
MSH3	MutS Homologue 3
MSH6	MutS Homologue 6
PMS2	Postmeiotic Segregation Increased 2
*KRAS*	V-Ki-ras2 Kirsten rat sarcoma viral oncogene homolog
*NRAS*	Neuroblastoma RAS viral oncogene homolog
*BRAF*	v-Raf murine sarcoma viral oncogene homolog B1
IQR	Interquartile range
OS	Overall survival
PFS	Progression-free survival
CR	Complete response
PR	Partial response
SD	Stable disease
PD	Progressive disease
NE	Not evaluable
ORR	Overall response rate
DCR	Disease control rate
VAF	Variant allele frequency
bTMB	Blood tumor mutational burden
TF	Tumor fraction
CI	Confidence interval
HR	Hazard ratio
SD	Standard deviation
CGP	Comprehensive genomic profiling

## Appendix A. Discordant Cases and Access and Response to Immunotherapy, *n* = 32

**ID**	**Issue Type**	**IHC**	**Molecular**	**ctMSI**	**ctCGP**	**Germline Pathogenic** **MMR Variant**	**Status**	**ICI**	**ICI** **Response**
	Tissue testing discordance								
1	IHC vs. molecular assay	pMMR	MSI-high	Not tested	Not tested	Not tested	MSI	Yes	CR
2	Uncommon IHC (equivocal staining and uncommon pairing)	Equivocal-MSH2/PMS2 (not confirmed)	Not tested	Not tested	Not tested	Not tested	MSS	No	
3	Uncommon IHC (equivocal staining)	Equivocal-MSH2 (not confirmed)	Not tested	ctMSS	None	Not tested	MSS	No	
4	Uncommon IHC (equivocal staining)	Equivocal-PMS2	MSS	ctMSS	Not tested	None	MSS	No	
5	Uncommon IHC (equivocal staining)	Equivocal-PMS2 (not confirmed)	Not tested	Not tested	Not tested	Not tested	MSS	No	
6	Uncommon IHC (equivocal staining)	Heterogenous MLH1	MSS	ctMSS	Not tested	Not tested	MSS	No	
7	Uncommon IHC (isolated loss and spatial heterogeneity)	dMMR-MSH6 (primary, not confirmed) pMMR (meta)	MSS	Not tested	Not tested	None	MSS	No	
8	Uncommon IHC (isolated loss and spatial heterogeneity)	pMMR (primary) dMMR-MSH6 (meta)	MSI-high	Not tested	Not tested	MSH6	MSI	Yes	CR
9	Uncommon IHC (isolated loss and spatial heterogeneity)	pMMR (primary) dMMR-PMS2 (meta)	MSI-high	ctMSS	None	Not tested	MSI	Yes	PR
10	Uncommon IHC (isolated loss)	dMMR-MLH1	Not tested	Not tested	Not tested	Not tested	MSI	No	
11	Uncommon IHC (isolated loss)	dMMR-MLH1	Not tested	Not tested	Not tested	Not tested	MSI	Yes	SD
12	Uncommon IHC (isolated loss)	dMMR-MSH6	MSI-high	Non-informative	Non-informative	MSH6	MSI	Yes	CR
13	Uncommon IHC (isolated loss)	dMMR-MSH6	MSI-high	Not tested	Not tested	None	MSI	Yes	CR
14	Uncommon IHC (isolated loss)	dMMR-MSH6	MSI-high	Not tested	Not tested	Not tested	MSI	No	
15	Uncommon IHC (isolated loss)	dMMR-MSH6 (not confirmed)	MSS	ctMSS	None	None	MSS	No	
16	Uncommon IHC (isolated loss)	dMMR-PMS2	MSI-high	ctMSI-high	None	Not tested	MSI	Yes	CR
17	Uncommon IHC (isolated loss)	dMMR-PMS2	MSI-high	ctMSS	PMS2 c.137G>T (VAF 46%)	PMS2	MSI	Yes	CR
18	Uncommon IHC (isolated loss)	dMMR-PMS2	MSI-high	Not tested	Not tested	Not tested	MSI	Yes	CR
19	Uncommon IHC (isolated loss)	dMMR-PMS2	MSI-low	Not tested	Not tested	Not tested	MSS	Yes	PD
20	Uncommon IHC (isolated loss)	dMMR-PMS2	MSS	ctMSS	None	Not tested	MSS	No	
21	Uncommon IHC (isolated loss)	dMMR-PMS2	MSS (Idylla) MSI-high (Pentaplex)	Not tested	Not tested	Not tested	MSI	No	
22	Uncommon IHC (spatial heterogeneity)	Equivocal-MLH1/PMS2 (primary) pMMR (meta)	Primary not tested Meta MSS	ctMSS	None	None	MSS	Yes	PD
23	Uncommon IHC (uncommon pairing)	dMMR-MLH1/MSH6	Not tested	Not tested	Not tested	Not tested	MSI	No	
	*Tissue vs. blood discordance*								
24	Blood missed	dMMR-MLH1/PMS2	MSI-high	ctMSS	None	None	MSI	No	
25	Blood missed	dMMR-MLH1/PMS2	MSI-high	ctMSS	None	None	MSI	Yes	SD
26	Blood missed	dMMR-MLH1/PMS2	MSI-high	ctMSS	None	Not tested	MSI	Yes	PD
27	Tissue missed	pMMR	Not tested	ctMSS	PMS2 W841* (VAF 29.9%)	Not tested	MSI	No	
28	Tissue missed	pMMR	MSS	ctMSI-high	Not tested	Not tested	MSI	No	
29	Tissue missed	pMMR	Not tested	ctMSS	MSH6 splice site 3801+1G>T (VAF 0.99%)	Not tested	MSI	No	
30	Tissue missed	pMMR	Not tested	ctMSS	None	MSH6	MSI	No	
31	Tissue missed	pMMR	Not tested	ctMSS	MLH1 S131* (VAF 1.4%)	Not tested	MSI	No	
32	Tissue missed	pMMR	Not tested	Not tested	Not tested	PMS2	MSI	No	
